# Ten-year retrospect of the investigation of proximal limbs metastasis in cancer: a multi-center study on survival outcome, limb function status and surgical procedures analysis

**DOI:** 10.1186/s12885-023-11292-5

**Published:** 2023-08-24

**Authors:** Chao Zhang, Jun Wang, Haixiao Wu, Yile Lin, Vladimir P. Chekhonin, Karl Peltzer, Artem V. Bukharov, Andrey D. Kaprin, Xu Guo, Zheng Liu

**Affiliations:** 1 Key Laboratory of Cancer Prevention and Therapy, Tianjin Medical University Cancer Institute and Hospital, National Clinical Research Center for Cancer, Tianjin’s Clinical Research Center for Cancer, Tianjin, China; 2 The Sino-Russian Joint Research Center for Bone Metastasis in Malignant Tumor, Tianjin, China; 3 Department of Oncology, Radiology and Nuclear Medicine, Medical Institute of Peoples’ Friendship University of Russia, Moscow, Russian Federation; 4 Department of Basic and Applied Neurobiology, Federal Medical Research Center for Psychiatry and Narcology, Moscow, Russian Federation; 5 Department of Psychology, University of the Free State, Turfloop, South Africa; 6 P.A. Hertsen Moscow Oncology Research Center - Branch of Federal State Budgetary Institution National Medical Research Radiological Center of the Ministry of Health of the Russian Federation, Moscow, Russia; 7 Department of Orthopedics, Cangzhou Central Hospital, Hebei province, Cangzhou, China; 8 Department of Orthopedics, Heilongjiang Province Hospital, Heilongjiang province, Harbin, China; 9 Department of Orthopedics, The Seventh Affiliated Hospital, Sun Yat-sen University, Guangdong province, Shenzhen, China; 10 Department of Bone and Soft Tissue Tumors, Tianjin Medical University Cancer Institute and Hospital, Tianjin, China

**Keywords:** Neoplasm Metastasis, Limb metastasis, Prognosis, Function evaluation

## Abstract

**Background:**

The aim of study was to evaluate survival outcome and limb function in cancer patients with proximal limbs metastasis. Associated factors on survival outcome and limb function were identified. The comparative analysis between intramedullary nailing and prosthesis surgery in cancer patients with proximal limb metastasis was performed.

**Methods:**

In this five-center retrospective study, patients diagnosed with limbs metastasis were collected. Descriptive statistics was used and log-rank test was performed to analyze the survival in subgroups. The Cox proportional hazards regression analysis was performed to identify the independent prognostic factors. The Musculoskeletal Tumor Society (MSTS) scoring system was used to evaluate limb function after surgery, and t test or analysis of variance (ANOVA) was utilized in subgroup analysis.

**Results:**

A total of 316 patients with limb metastasis were included with mean age at 61.0 years. The most common primary tumor was breast, followed by renal cancer and lung cancer. The median overall survival was 24.0 months and the 1-, 3- and 5-year survival rates were 86.9%, 34.7% and 6.8%, respectively. Primary tumor type, visceral metastasis and chemotherapy were proved to be the independent prognostic factors. The mean Musculoskeletal Tumor Society (MSTS) score was 20.5, significant difference was observed in subgroup of solitary/multiple bone metastasis, with/without pathological fracture, and type of surgery.

**Conclusion:**

The present study concluded that primary tumor type, visceral metastasis and chemotherapy were three factors affecting the survival of patients. Compared with intramedullary nailing, the patients underwent prosthesis surgery showed better limb function, this procedure should be encouraged in patients with indication.

**Supplementary Information:**

The online version contains supplementary material available at 10.1186/s12885-023-11292-5.

## Introduction

Due to the advances in treatment strategies and improvement in diagnosis, the prevalence of bone metastasis (BM) has been increasing in recent years [1, 2]. It was reported that BM occurred in approximately 70% of cancer patients with metastatic disease [3, 4]. As most tumors predominantly metastasize to axial skeleton, literatures on spinal metastasis were widely reported [5–8]. Limbs metastasis accounted for 10% of patients with BM, the study addressing on patients with limbs metastasis was limited [9]. However, limbs metastasis can significantly reduce the patients’ quality of life.

Breast cancer, prostate cancer, renal cell carcinoma and lung cancer are common primary tumors resulting in limbs metastasis [1, 10–13]. Previous literatures reported that limbs metastasis can be found in some unusual cancers such as endometrial carcinoma and Merkel cell carcinoma [14, 15]. The proximal long bone including humerus and femur was the most common site in limbs metastasis [16]. Other uncommon metastatic sites such as tibia were also reported [17]. The 5-year survival rates of patients with limbs metastasis ranged from 8.0 to 64.3% due to different cohort selection [18]. The survival rate of breast cancer was up to more than 50% while that of lung cancer was less than 25% [19]. A retrospective study included 114 cancer patients with limbs metastasis and concluded that primary tumor, visceral metastasis and surgical procedure were associated with survival outcome [20]. Limited clinical variables were involved in the study, which restricted the finding of the study [20]. The survival estimation of cancer patients usually determined the choice of the individual treatment [21–23]. Thus, further study is warrant.

With the development of bone target therapy and comprehensive treatment on primary cancer, the aim of the treatment in cancer patients with limbs metastasis has been turning into the integration of symptom palliation and function improvement [24]. Patients with limbs metastasis usually suffered from refractory pain, pathological fracture, limited mobility and emotion damage, which significantly reduced patients’ quality of life and increased patients’ medical costs [25]. Limb function has been receiving attention from both patients and physicians. Several scoring systems were reported on evaluating limb function, including Musculoskeletal Tumor Society (MSTS) scoring system, Toronto Extremity Salvage Score (TESS) [26, 27].

For cancer patients with limbs metastasis, systematic chemotherapy on primary tumor was cornerstone and radiation therapy was usually recommended to relief pain [4]. Surgical intervention was encouraged for patients with high risk of bone fracture and clear pathological fracture. Prosthesis and intramedullary nailing have been accepted to be two main strategies on bone structure reconstruction [28]. Based on minimally invasive surgical techniques, percutaneous treatments such as ablation (thermal ablation, cryoablation and high intensity radiofrequency ablation) and cementoplasty were also performed in selected patients for palliative purposes [29]. The choice of the surgical method is still in controversial.

Based on data from multi-centers, the present study conducted a comprehensive analysis aiming to assess the survival outcome of cancer patients with limbs metastasis. Moreover, limb function was evaluated and compared among different subgroups. Our study can potentially help physicians estimate the prognosis and limb function of cancer patients with limbs metastasis, and tailor targeted treatment regimens.

## Materials and methods

### Data sources and study population

Conducting based on *The Sino-Russian Joint Research Center for Bone Metastasis in Malignant Tumor, Tianjin, China*, the data of initial included patients were derived from five centers from China and Russia as following: (1) Cangzhou Central Hospital, Cangzhou, Hebei Province, China; (2) Heilongjiang Provincial Hospital, Harbin, Heilongjiang Province, China; (3) P.A. Herzen Moscow Oncology Research Institute, Branch, National Medical Radiology Research Center, Ministry of Health Russia, Moscow, Russia; (4) MRNC named after A.F. Tsyba - a branch of the FSBI National Medical Research Center for Radiology of the Ministry of Health of Russia, Obninsk, Russia; (5) Institute of Medicine, Peoples’ Friendship University of Russia; Moscow, Russia. This retrospective, multi-centers study was conducted in accordance with the 1975 Helsinki Declaration and its later amendments or comparable ethical standards and was approved by the Ethics Board of the P.A. Hertsen Moscow Oncology Research Center (CT3772Y4J).

**Fig. 1:**
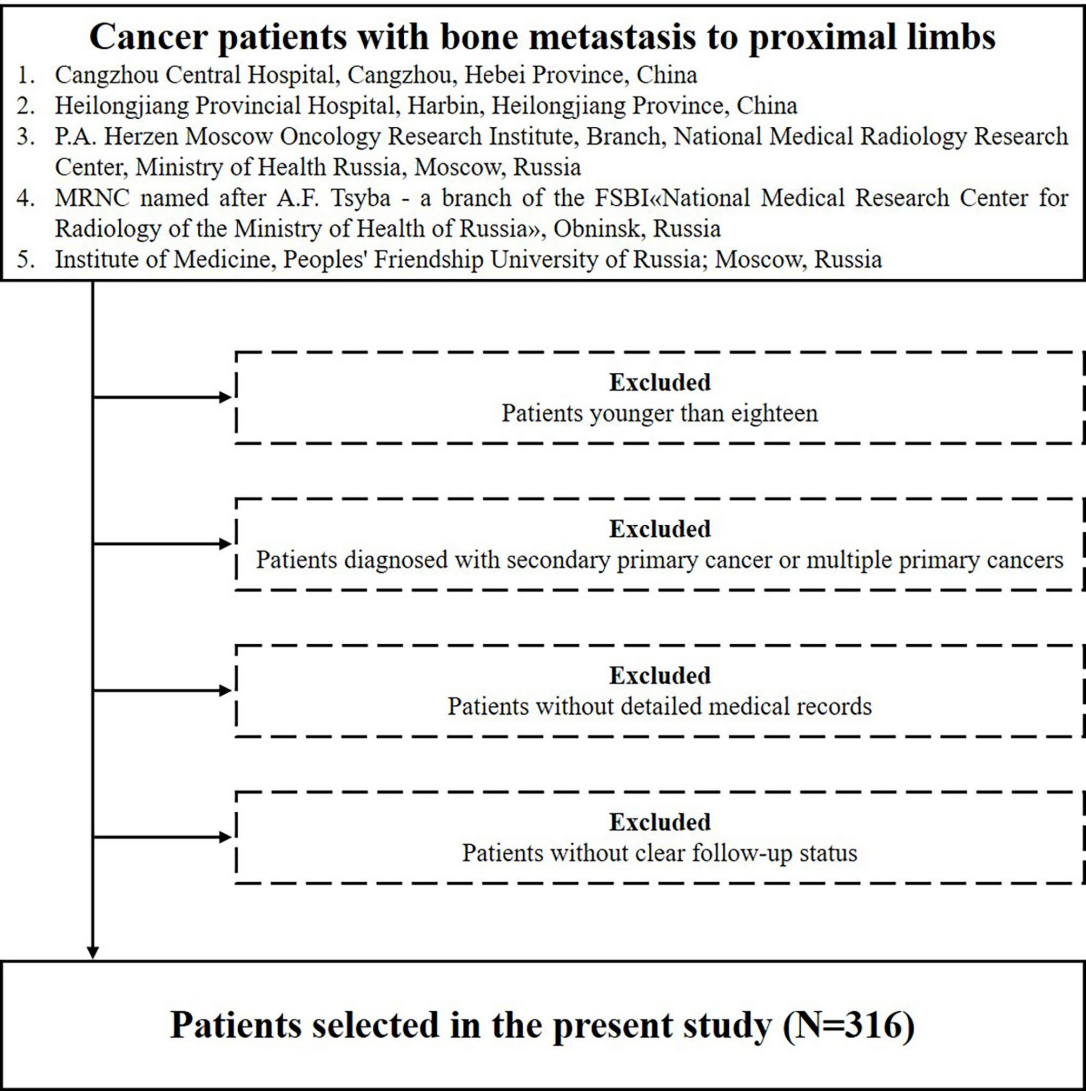
The flowchart of the patient selection in the study

The present study preliminarily included patients who were diagnosed with bone metastasis to proximal limbs (humerus or femur). The diagnosis of limb metastasis was confirmed by image examinations including X-ray, computed tomography (CT) scan, magnetic resonance imaging (MRI), bone scintigraphy and positron emission tomography (PET) or confirmed by pathology examinations. The exclusion criteria were as following: (1) patients younger than eighteen; (2) patients diagnosed with secondary primary cancer or multiple primary cancers; (3) patients without detailed medical records; (4) patients without clear follow-up status. The flowchart of the patient selection was shown in Fig. 1.

### Study variables

The following medical records were collected and evaluated: age of diagnosis, gender (male or female), preoperative BMI, smoking or alcohol consumption (no or yes), ABO blood type (A type, B type, AB type or O type), T-stage for primary cancer (T1, T2, T3 or T4), lymph metastasis (no or yes), visceral metastasis (no or yes), bone metastasis (solitary or multiple), pathological fracture (no or yes), surgical anatomic location (humerus or femur), type of surgery (prosthesis or intramedullary nailing), days from diagnosis of limb metastasis to surgery, hospitalization days after surgery, duration of surgery, intraoperative blood loss, blood transfusion (no or yes), adjuvant chemotherapy (no or yes), adjuvant radiotherapy (no or yes), Musculoskeletal Tumor Society (MSTS) score. Primary tumor type was defined by pathology and classified into three groups according to tumor malignancy [20, 30].

### Statistical analysis

Quantitative data were described as mean ± standard error of the mean (SEM) and independent sample *t* test or analysis of variance (ANOVA) was utilized to find the difference among subgroups. Categorical variables were presented as number and percentage (N, %). Pearson chi-square (χ2) test or Fisher’s exact test was used to evaluate the difference. The primary outcome in the present study was overall survival (OS), which was defined as the interval between the diagnosis of limb metastasis and all causes of death. Kaplan-Meier curves and log-rank tests were used to analyze survival difference. The Cox proportional hazards regression analysis was performed to identify the independent prognostic factors. The IBM SPSS Statistics (version 26.0, Armonk, NY, USA) was used for statistical analysis. All statistical tests were two-sided, and *P < 0.05* was considered significant.

## Results

### Baseline characteristics of the patients

A total of 316 cancer patients with proximal limbs metastasis were retrospectively included. The mean age at initial diagnosis was 61.0 ± 0.6 years with a slightly female predominance (N = 194, 61.4%). 53.8% (N = 170) of patients presented solitary bone metastasis while others (N = 146, 46.2%) presented multiple bone metastasis. Pathological fracture occurred in 212 patients (67.1%). Adjuvant chemotherapy and adjuvant radiotherapy was performed in 203 (64.2%) and 122 (38.6%) cases, respectively. In the total cohort, 103 patients (32.6%) underwent surgery on humerus while 213 cases (67.4%) underwent surgery on femur. More information about baseline characteristics of patients with limb metastasis was shown in Table 1.

**Table 1:** Baseline characteristics of patients with proximal limb metastasis in the present study

Characteristic	Surgical anatomic location		Total cohort (N = 316)
	Humerus (N = 103)	Femur (N = 213)	
**Age (years)**	61.4 ± 1.1	60.8 ± 0.7	61.0 ± 0.6
**BMI (kg/m** ^**2**^ **)**	25.4 ± 0.4	25.7 ± 0.3	25.6 ± 0.2
**Gender**			
Male	46 (44.7%)	76 (35.7%)	122 (38.6%)
Female	57 (55.3%)	137 (64.3%)	194 (61.4%)
**Primary tumor**			
Slow growth	45 (43.7%)	103 (48.4%)	148 (46.8%)
Moderate growth	25 (24.3%)	56 (26.3%)	81 (25.6%)
Rapid growth	33 (32.0%)	54 (25.4%)	87 (27.5%)
**Smoking or alcohol consumption**			
No	73 (70.9%)	154 (72.3%)	227 (71.8%)
Yes	20 (19.4%)	51 (23.9%)	71 (22.5%)
Unknown	10 (9.7%)	8 (3.8%)	18 (5.7%)
**ABO blood type**			
A type	38 (36.9%)	63 (29.6%)	101 (32.0%)
B type	24 (23.3%)	67 (31.5%)	91 (28.8%)
AB type	13 (12.6%)	24 (11.3%)	37 (11.7%)
O type	28 (27.2%)	59 (27.7%)	87 (27.5%)
**T stage for primary tumor**			
T1	12 (11.7%)	21 (9.9%)	33 (10.4%)
T2	42 (40.8%)	114 (53.5%)	156 (49.4%)
T3	37 (35.9%)	52 (24.4%)	89 (28.2%)
T4	12 (11.7%)	26 (12.2%)	38 (12.0%)
**Lymph metastasis**			
No	46 (44.7%)	110 (51.6%)	156 (49.4%)
Yes	57 (55.3%)	103 (48.4%)	160 (50.6%)
**Visceral metastasis**			
No	76 (73.8%)	153 (71.8%)	229 (72.5%)
Yes	27 (26.2%)	60 (28.2%)	87 (27.5%)
**Bone metastasis**			
Solitary	59 (57.3%)	111 (52.1%)	170 (53.8%)
Multiple	44 (42.7%)	102 (47.9%)	146 (46.2%)
**Pathological fracture**			
No	34 (33.0%)	70 (32.9%)	104 (32.9%)
Yes	69 (67.0%)	143 (67.1%)	212 (67.1%)
**Type of surgery**			
Prosthesis	74 (71.8%)	149 (70.0%)	223 (70.6%)
Intramedullary Nailing	29 (28.2%)	64 (30.0%)	93 (29.4%)
**Blood transfusion**			
No	83 (80.6%)	169 (79.3%)	252 (79.7%)
Yes	20 (19.4%)	44 (20.7%)	64 (20.3%)
**Days from diagnosis to surgery (d)**	5.7 ± 0.3	8.0 ± 0.3	7.3 ± 0.2
**Hospitalization days after surgery (d)**	11.4 ± 0.3	11.2 ± 0.2	11.3 ± 0.2
**Duration of surgery (min)**	155.7 ± 4.3	177.8 ± 4.2	170.6 ± 3.2
**Intraoperative blood loss (ml)**	508.7 ± 58.0	623.1 ± 52.3	585.4 ± 39.9
**Adjuvant chemotherapy**			
No	36 (35.0%)	77 (36.2%)	113 (35.8%)
Yes	67 (65.0%)	136 (63.8%)	203 (64.2%)
**Adjuvant radiotherapy**			
No	65 (63.1%)	129 (60.6%)	194 (61.4%)
Yes	38 (36.9%)	84 (39.4%)	122 (38.6%)

**Fig. 2:**
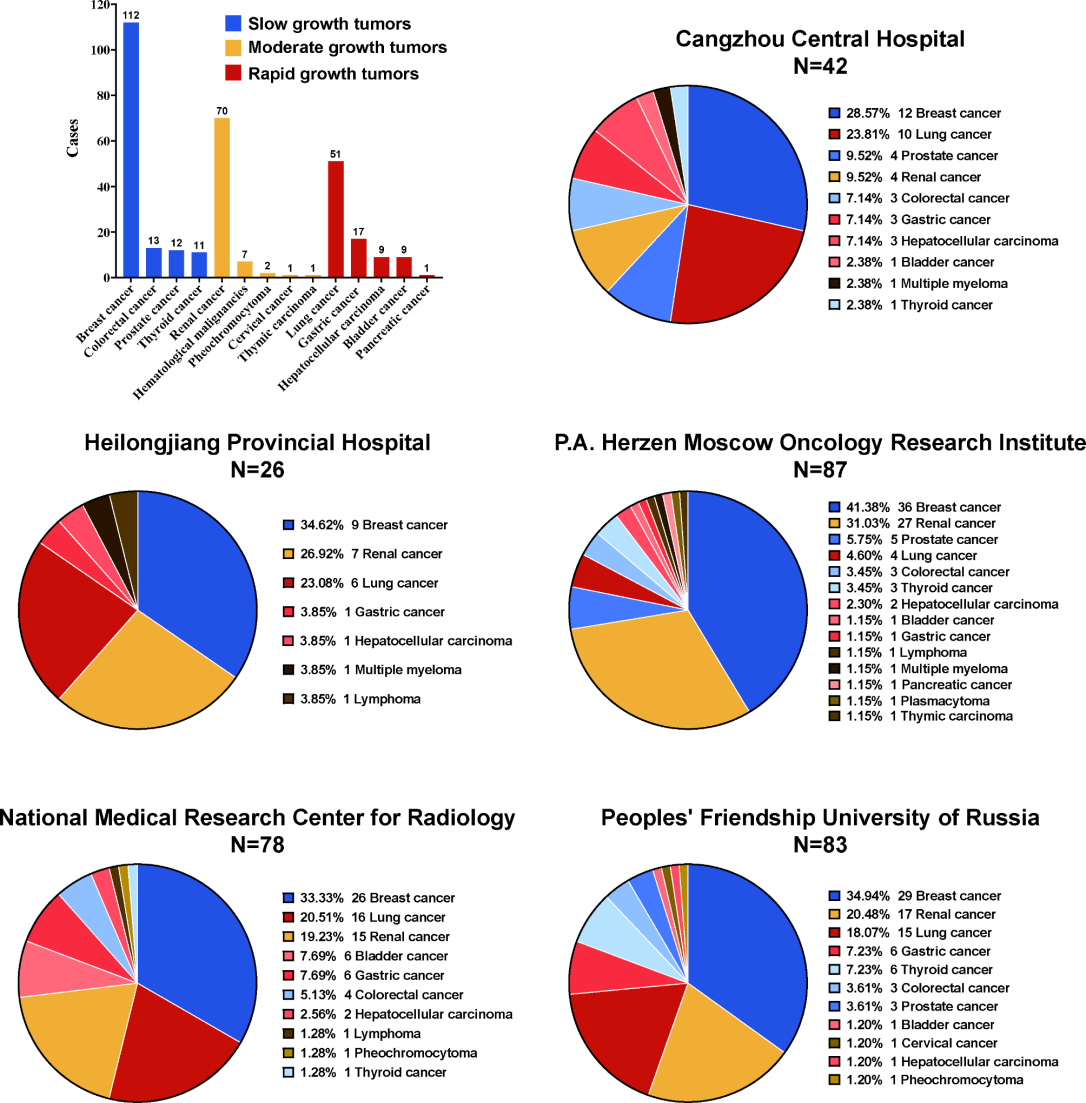
The number of cases and percentage of primary tumor in each clinical center

Primary tumor type was classified into three subgroups according to tumor malignancy: slow growth, moderate growth and rapid growth. Slow growth subgroups included breast cancer, prostate cancer, thyroid cancer and colorectal cancer. Moderate growth included renal cancer, thymic carcinoma, cervical cancer, pheochromocytoma and hematological malignant tumor (three for multiple myeloma, three for lymphoma and one for plasmacytoma). Rapid growth included hepatocellular carcinoma, lung cancer, gastric cancer, bladder cancer and pancreatic cancer. Breast cancer (N = 112), renal cancer (N = 70) and lung cancer (N = 51) were the major primary cancer types in the total cohort and were the most common type in subgroup of slow growth, moderate growth and rapid growth, respectively. The number of cases in each center were listed as following: 42 of Cangzhou Central Hospital; 26 of Heilongjiang Provincial Hospital; 87 of P.A. Herzen Moscow Oncology Research Institute; 78 of National Medical Research Center for Radiology and 83 of Peoples’ Friendship University of Russia. The number of cases and percentage of primary tumor in each center were shown in Fig. 2.

### Survival outcome and prognostic factors

**Fig. 3:**
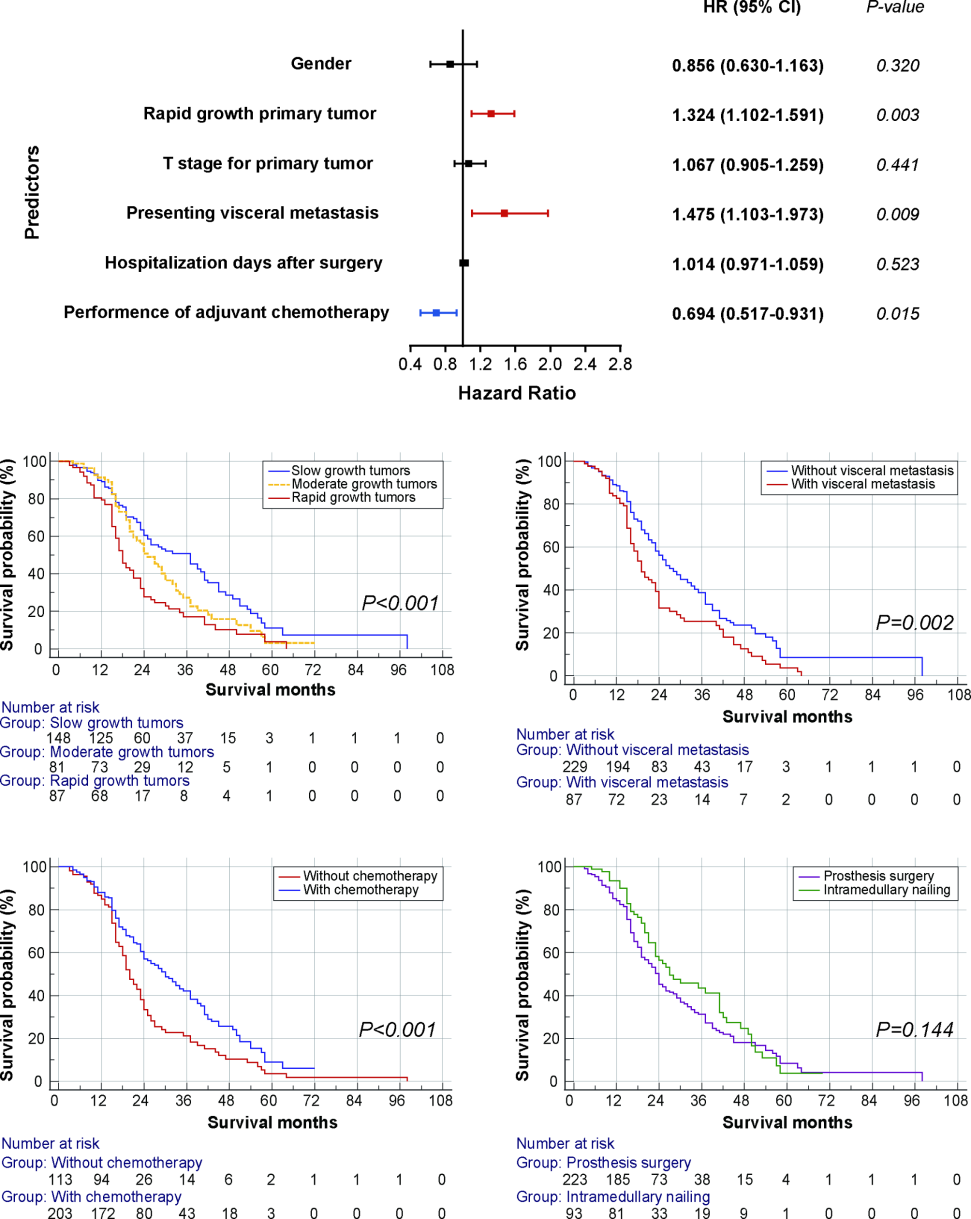
The forest plot of Cox proportional hazards regression analysis and survival curves in subgroups analyses

A total of 211 patients deceased at the last follow-up and the median OS was 24.0 (95% CI: 21.6–26.4) months. The 1-, 3- and 5-year survival rates were 86.9%, 34.7% and 6.8%, respectively. The survival outcome in each clinical center was described in Supplementary Table 1. As shown in Table 2, gender, primary tumor, T stage, visceral metastasis, hospitalization days and adjuvant chemotherapy were associated with survival in univariate Cox regression analysis. After adjusting these characteristics in multivariate analysis, rapid growth tumor (versus slow growth tumor, HR = 1.67, 95% CI: 1.15–2.42, *P = 0.007*), presenting visceral metastasis (versus no visceral metastasis, HR = 1.44, 95% CI: 1.08–1.94, *P = 0.014*) and no performance of adjuvant chemotherapy (versus adjuvant chemotherapy, HR = 0.69, 95% CI: 0.51–0.93, *P = 0.013*) were three independent prognostic factors for worse survival. More details about Cox proportional hazards regression analysis and survival curves in subgroups analyses were presented in Fig. 3.

**Table 2:** Cox proportional hazard regression model for analyzing the prognostic factors for malignant cancer patients with proximal limb metastasis

Subject characteristics	Univariate		Multivariate	
	HR (95% CI)	*P-value*	HR (95% CI)	*P-value*
**Age**	1.01 (1.00-1.02)	*0.118*	-	*-*
**BMI**	0.99 (0.95–1.04)	*0.703*	-	*-*
**Gender**				
Male	1 (reference)	*1.00*	1 (reference)	*1.00*
Female	0.70 (0.53–0.92)	*0.010*	0.90 (0.66–1.22)	*0.483*
**Primary tumor**				
Slow growth	1 (reference)	*1.00*	1 (reference)	*1.00*
Moderate growth	1.38 (0.99–1.94)	*0.061*	1.26 (0.88–1.80)	*0.205*
Rapid growth	2.08 (1.51–2.87)	*< 0.001*	1.67 (1.15–2.42)	*0.007*
**Smoking or alcohol consumption**				
No	1 (reference)	*1.00*	-	
Yes	1.23 (0.88–1.73)	*0.224*	-	*-*
Unknown	1.01 (0.57–1.78)	*0.973*	-	*-*
**ABO blood type**				
A type	1 (reference)	*1.00*	-	
B type	1.11 (0.79–1.56)	*0.565*	-	*-*
AB type	1.43 (0.90–2.26)	*0.130*	-	*-*
O type	0.96 (0.67–1.38)	*0.831*	-	*-*
**T stage for primary tumor**				
T1	1 (reference)	*1.00*	1 (reference)	*1.00*
T2	1.12 (0.67–1.86)	*0.661*	1.04 (0.62–1.74)	*0.892*
T3	1.96 (1.17–3.29)	*0.010*	1.42 (0.83–2.43)	*0.199*
T4	1.29 (0.71–2.33)	*0.406*	1.04 (0.56–1.91)	*0.913*
**Lymph metastasis**				
No	1 (reference)	*1.00*	-	
Yes	0.90 (0.69–1.18)	*0.451*	-	*-*
**Visceral metastasis**				
No	1 (reference)	*1.00*	1 (reference)	*1.00*
Yes	1.58 (1.19–2.09)	*0.002*	1.44 (1.08–1.94)	*0.014*
**Bone metastasis**				
Solitary	1 (reference)	*1.00*	-	
Multiple	0.84 (0.64–1.10)	*0.211*	-	*-*
**Pathological fracture**				
No	1 (reference)	*1.00*	-	
Yes	0.97 (0.73–1.30)	*0.857*	-	*-*
**Surgical anatomic location**				
Humerus	1 (reference)	*1.00*	-	
Femur	0.89 (0.67–1.18)	*0.420*	-	*-*
**Type of surgery**				
Prosthesis	1 (reference)	*1.00*	-	
Intramedullary Nailing	0.79 (0.58–1.08)	*0.143*	-	*-*
**Blood transfusion**				
No	1 (reference)	*1.00*	-	
Yes	0.92 (0.65–1.31)	*0.651*	-	*-*
**Days from diagnosis to surgery**	0.99 (0.96–1.03)	*0.721*	-	*-*
**Hospitalization days after surgery**	1.04 (1.00-1.09)	*0.036*	1.01 (0.97–1.06)	*0.566*
**Duration of surgery**	1.00 (1.00–1.00)	*0.432*	-	*-*
**Intraoperative blood loss**	-	*0.117*	-	*-*
**Adjuvant chemotherapy**				
No	1 (reference)	*1.00*	1 (reference)	*1.00*
Yes	0.62 (0.47–0.82)	*0.001*	0.69 (0.51–0.93)	*0.013*
**Adjuvant radiotherapy**				
No	1 (reference)	*1.00*	-	
Yes	0.87 (0.65–1.16)	*0.338*	-	*-*
**MSTS score**	1.00 (0.93–1.07)	*0.891*	-	*-*

### Musculoskeletal Tumor Society (MSTS) score and limb function evaluation

The mean Musculoskeletal Tumor Society (MSTS) score was 20.50 ± 0.111. As shown in Table 3, the MSTS score of solitary bone metastatic patients (20.91 ± 0.137) was significantly higher than that of multiple bone metastatic patients (19.96 ± 0.169, *P < 0.001*). Patients presenting pathological fracture showed lower MSTS score (20.26 ± 0.129) than those without pathological fracture (20.88 ± 0.204, *P = 0.008*). Compared with patients underwent intramedullary nailing surgery (19.41 ± 0.195), patients who underwent prosthesis surgery presented higher MSTS score (20.91 ± 0.123, *P < 0.001*). Meanwhile, the baseline characteristics of patients grouped by type of surgery were listed in Supplementary Table 2.

**Table 3:** Musculoskeletal Tumor Society (MSTS) score in patients with proximal limb metastasis

Subject characteristics	Mean	SEM	F/t	*P-value*
**Total cohort**	20.50	0.111	*-*	*-*
**Gender**				
Male	20.29	0.179	-1.302	*0.194*
Female	20.58	0.141		
**Primary tumor**				
Slow growth	20.53	0.166	3.379	*0.035*
Moderate growth	20.81	0.222		
Rapid growth	20.05	0.191		
**Smoking or alcohol consumption**				
No	20.59	0.128	1.687	*0.187*
Yes	20.13	0.237		
Unknown	20.22	0.515		
**ABO blood type**				
A type	20.64	0.198	0.415	*0.742*
B type	20.37	0.218		
AB type	20.32	0.333		
O type	20.43	0.194		
**T stage for primary tumor**				
T1	20.36	0.350	0.922	*0.431*
T2	20.47	0.168		
T3	20.67	0.196		
T4	20.05	0.258		
**Lymph metastasis**				
No	20.44	0.158	-0.232	*0.817*
Yes	20.49	0.155		
**Visceral metastasis**				
No	20.47	0.123	-0.015	*0.988*
Yes	20.47	0.241		
**Bone metastasis**				
Solitary	20.91	0.137	4.389	*< 0.001*
Multiple	19.96	0.169		
**Pathological fracture**				
No	20.88	0.204	2.660	*0.008*
Yes	20.26	0.129		
**Surgical anatomic location**				
Humerus	20.64	0.191	1.084	*0.279*
Femur	20.38	0.136		
**Type of surgery**				
Prosthesis	20.91	0.123	6.588	*< 0.001*
Intramedullary Nailing	19.41	0.195		

## Discussion

As retrospective study reported, a total of 539 patients diagnosed with BM in the University of Tokyo Hospital were categorized into four groups according to bone metastatic sites [31]. Patients with metastasis to humerus or femur were defined as ‘rhizometastasis’. The study concluded that the patients with rhizometastasis accounted for 22.5% with the median survival of 16.0 months [31]. As a note, there were several differences between humerus metastasis and femur metastasis despite of their similarities. Femur metastasis was much more common in clinic while humerus metastasis was relatively rare, especially as isolated or initial metastatic site [32, 33]. In a retrospectively study focusing on metastasis in long bones, the proportion of patients suffered from femur metastasis was up to 73.7% (84 cases) while the proportion was 22.8% in humerus metastasis [34]. Considering the weight-bearing role of femur, compared with humerus metastasis, the quality of life can be more determined by femur metastasis. Thus, among the bone metastasis patients with limited survival, the active skeletal reconstruction was usually suggested to the patients with femur pathological fracture, while the palliative fixation brace can be given to the patients with humerus pathological fracture. As for primary tumor types, another study included 164 patients with limb metastasis and the top five common primary tumor lesions were, in descending order, breast, lung, renal, prostate and myeloma [35]. Being different from the above-mentioned studies with single center data, we performed an international investigation and reported 316 patients derived from five cohorts in China and Russia. The baseline characteristics of patients were summarized and described in humerus and femur respectively (shown in Table 1), and survival outcome and limb function were evaluated. Breast cancer, renal cancer and lung cancer were proved to be the most common primary tumors while different survival outcomes were confirmed between subgroups.

As for patients with spinal metastasis, primary tumor was identified as the fundamental prognostic predictor in several well-established score models including Tomita score, Tokuhashi score and Linden score [30]. Similarly, multiple studies confirmed the impact of primary tumor on prognosis in patients with limbs metastasis [16, 20]. A study, including a total of 301 limbs metastatic patients, concluded that breast or prostate was associated with a better survival outcome compared with other primary lesions [16]. The postoperative survival was up to 16.0 months and 17.0 months in breast cancer and prostate cancer, respectively. The survival ranged from 4.0 to 9.0 months in more aggressive tumors such as lung cancer, bladder cancer and renal cell carcinoma [16]. Based on the analysis of 102 patients with upper extremity metastasis, another study demonstrated that there was a 4.4-fold increased risk of death in patients with rapid growth primary tumor [36]. It was reported that primary tumor was a risk factor for 30-day postoperative complications in patients with limb metastasis, and the complication rate was associated with 1-year mortality after surgery [37].

Compared to bone metastasis, visceral metastasis was generally accepted to be associated with overall cancer mortality in patients with advanced-stage cancer. The current medical therapies for visceral metastasis were inconsistent and limited despite of the recent therapeutic advances, and this metastatic disease was thought to be irreversible and incurable [38, 39]. Lung, liver and brain were regarded as the major target sites of visceral metastasis and patients with these metastatic organs always suffered from malignant pleural effusion, dysfunction of liver, headache and focal neurological deficits [40]. Based on SEER database, a total of 12,794 prostate cancer patients with BM were included and prognostic factors were identified [41]. The presence of lung, liver and brain metastasis were three predictive factors for worse survival outcome in bone metastatic prostate cancer [41]. There were significant differences of median survival between patients with or without visceral metastasis [41]. In the current study, visceral metastasis was retrospectively analyzed and there was a 1.48-fold increased risk of death for patients with visceral metastasis, which was consistent with previous studies [40–43]. Besides, the total cohort benefited on survival from the performance of chemotherapy with a 0.7-fold increased risk of death in our study. Except for anti-tumor role of chemotherapy, the potential cause could be better performance status, more family supports and higher economic possess in patients received systematic chemotherapy.

Musculoskeletal Tumor Society (MSTS) scoring system was widely accepted to measure limb function after surgery in oncological surgeon. The scoring system was originally developed in 1985 and subsequently adopted in 1993 [26]. MSTS scoring system comprised six categories (0–5 points for each category) and higher total points indicated better limb function[26]. Three categories were fitted for both upper and lower extremities: pain, function, emotional acceptance. While supports, walking ability and gait were for the lower extremity; and hand positioning, dexterity and lifting ability were used for the upper extremity. Our study concluded that patients with multiple bone metastatic sites and pathological fracture presented lower total points, which could be on account of impaired mobility and emotional distress. In the current study, patients who underwent surgery of intramedullary nailing presented lower MSTS score than patients with prosthesis surgery. Usually, these two surgical procedures were selected according to the metastatic tumor site. Prosthesis surgery was used on patients with tumor adjacent to the joint while intramedullary nailing on patients with fracture located in the backbone [9]. To compare the surgical outcome between intramedullary nailing and modular megaprosthesis, forty-five patients with extracapsular metastases of proximal femur were retrospectively collected [44]. The shorter operative time and more rapid functional recovery were observed in intramedullary group [44]. In addition, previous studies suggested a lower complication rate and shorter hospitalization in patients received intramedullary nailing [28, 45]. However, our study revealed the further functional benefit from prosthesis surgery when compared with intramedullary nailing. Thus, prosthesis surgery should be encouraged on the patients with the clear indication.

The study was performed retrospectively, thus inherent selection bias was hardly avoided. Besides, only MSTS scoring system was used to evaluate limb function. Other scoring systems such as Toronto Extremity Salvage Score (TESS) could be used in future study.

## Conclusions

The present study provided a perspective on survival outcome and limb function in cancer patients with limb metastasis. Primary tumor type, visceral metastasis and systematic chemotherapy treatment were the independent prognostic factors. Better limb function after surgery was seen in patients with solitary bone metastasis and those without pathological fracture. Compared with intramedullary nailing, patient’s limb function can further benefit from prosthesis surgery. As for patients with limbs metastasis, our findings can be potentially used in survival estimation and individualized treatment planning generation.

### Electronic supplementary material

Below is the link to the electronic supplementary material.

Supplementary Material 1

Supplementary Material 2

## Data Availability

The datasets used in the current study can be accessed from the corresponding author on reasonable request.

## List of abbreviations

MSTS: Musculoskeletal Tumor Society
BM: Bone Metastasis
TESS: Toronto Extremity Salvage Score
CT: Computed Tomography
MRI: Magnetic Resonance Imaging
PET: positron emission tomography
OS: overall survival
