# Supplementary material for: Ten-year retrospect of the investigation of proximal limbs metastasis in cancer: a multi-center study on survival outcome, limb function status and surgical procedures analysis
Source: BMC Cancer. 2023 Aug 24;23:795. doi: 10.1186/s12885-023-11292-5 (PMC10463808; doi:10.1186/s12885-023-11292-5)
Supplement: Supplementary file 1 — Supplementary Material 1 [file 12885_2023_11292_MOESM1_ESM.docx]

**Supplementary Table 1. The survival outcome in each clinical center.**

| **Center** | **Number of deaths** | **Median OS (Months)** | **1-year OS rate** | **3-year OS rate** | **5-year OS rate** |
| --- | --- | --- | --- | --- | --- |
| **Cangzhou Central Hospital** | 26 | 23.0 (95% CI: 20.2-25.8) | 95.1% | 42.3% | 13.2% |
| **Heilongjiang Provincial Hospital** | 20 | 17.0 (95% CI: 12.5-21.5) | 80.6% | 28.2% | 0% |
| **P.A. Herzen Moscow Oncology Research Institute** | 70 | 23.0 (95% CI: 18.0-28.0) | 73.6% | 30.6% | 9.8% |
| **National Medical Research Center for Radiology** | 48 | 24.0 (95% CI: 20.9-27.1) | 93.5% | 29.6% | 4.5% |
| **Peoples' Friendship University of Russia** | 47 | 28.0 (95% CI: 18.2-37.8) | 92.7% | 42.3% | 5.6% |
