# Supplementary material for: Ten-year retrospect of the investigation of proximal limbs metastasis in cancer: a multi-center study on survival outcome, limb function status and surgical procedures analysis
Source: BMC Cancer. 2023 Aug 24;23:795. doi: 10.1186/s12885-023-11292-5 (PMC10463808; doi:10.1186/s12885-023-11292-5)
Supplement: Supplementary file 2 — Supplementary Material 2 [file 12885_2023_11292_MOESM2_ESM.docx]

**Supplementary Table 2. Baseline characteristics of the patients with proximal limb metastasis in the present study, grouped according to type of surgery.**

| **Characteristic** | **Type of surgery** | | **Total cohort**  **(N=316)** |
| --- | --- | --- | --- |
|  | **Prosthesis**  **(N=223)** | **Intramedullary Nailing**  **(N=93)** |  |
| **Age (years)** | 61.6±0.7 | 59.5±1.0 | 61.0±0.6 |
| **BMI (kg/m^2^)** | 26.9±0.3 | 24.8±0.4 | 25.6±0.2 |
| **Gender** |  |  |  |
| Male | 91 (40.8%) | 31 (33.3%) | 122 (38.6%) |
| Female | 132 (59.2%) | 62 (66.7%) | 194 (61.4%) |
| **Primary tumor** |  |  |  |
| Slow growth | 103 (46.2%) | 45 (48.4%) | 148 (46.8%) |
| Moderate growth | 58 (26.0%) | 23 (24.7%) | 81 (25.6%) |
| Rapid growth | 62 (27.8%) | 25 (26.9%) | 87 (27.5%) |
| **Smoking or alcohol consumption** |  |  |  |
| No | 159 (71.3%) | 68 (73.1%) | 227 (71.8%) |
| Yes | 48 (21.5%) | 23 (24.7%) | 71 (22.5%) |
| Unknown | 16 (7.2%) | 2 (2.2%) | 18 (5.7%) |
| **ABO blood type** |  |  |  |
| A type | 66 (29.6%) | 35 (37.6%) | 101 (32.0%) |
| B type | 64 (28.7%) | 27 (29.0%) | 91 (28.8%) |
| AB type | 26 (11.7%) | 11 (11.8%) | 37 (11.7%) |
| O type | 67 (30.0%) | 20 (21.5%) | 87 (27.5%) |
| **T stage for primary tumor** |  |  |  |
| T1 | 19 (8.5%) | 14 (15.1%) | 33 (10.4%) |
| T2 | 106 (47.5%) | 50 (53.8%) | 156 (49.4%) |
| T3 | 70 (31.4%) | 19 (20.4%) | 89 (28.2%) |
| T4 | 28 (12.6%) | 10 (10.8%) | 38 (12.0%) |
| **Lymph metastasis** |  |  |  |
| No | 114 (51.1%) | 42 (45.2%) | 156 (49.4%) |
| Yes | 109 (48.9%) | 51 (54.8%) | 160 (50.6%) |
| **Visceral metastasis** |  |  |  |
| No | 161 (72.2%) | 68 (73.1%) | 229 (72.5%) |
| Yes | 62 (27.8%) | 25 (26.9%) | 87 (27.5%) |
| **Bone metastasis** |  |  |  |
| Solitary | 132 (59.2%) | 38 (40.9%) | 170 (53.8%) |
| Multiple | 91 (40.8%) | 55 (59.1%) | 146 (46.2%) |
| **Pathological fracture** |  |  |  |
| No | 76 (34.1%) | 28 (30.1%) | 104 (32.9%) |
| Yes | 147 (65.9%) | 65 (69.9%) | 212 (67.1%) |
| **Surgical anatomic location** |  |  |  |
| Humerus | 74 (33.2%) | 29 (31.2%) | 103 (32.6%) |
| Femur | 149 (66.8%) | 64 (68.8%) | 213 (67.4%) |
| **Blood transfusion** |  |  |  |
| No | 188 (84.3%) | 64 (68.8%) | 252 (79.7%) |
| Yes | 35 (15.7%) | 29 (31.2%) | 64 (20.3%) |
| **Days from diagnosis to surgery (d)** | 6.8±0.3 | 8.2±0.3 | 7.3±0.2 |
| **Hospitalization days after surgery (d)** | 11.2±0.2 | 11.6±0.3 | 11.3±0.2 |
| **Duration of surgery (min)** | 172.0±3.8 | 167.3±6.1 | 170.6±3.2 |
| **Intraoperative blood loss (ml)** | 615.0±48.3 | 515.9±71.4 | 585.4±39.9 |
| **Adjuvant chemotherapy** |  |  |  |
| No | 73 (32.7%) | 40 (43.0%) | 113 (35.8%) |
| Yes | 150 (67.3%) | 53 (57.0%) | 203 (64.2%) |
| **Adjuvant radiotherapy** |  |  |  |
| No | 139 (62.3%) | 55 (59.1%) | 194 (61.4%) |
| Yes | 84 (37.7%) | 38 (40.9%) | 122 (38.6%) |
